# Trends in mortality, disability-adjusted life years, and years of healthy life lost due to self-harming in Brazilian states from 1990 to 2019

**DOI:** 10.11606/s1518-8787.2025059006322

**Published:** 2025-03-13

**Authors:** Milena Sabino Fonseca, Gustavo Gusmão dos Santos, Patricia Colombo de Souza, Lúcia Helena de Azevedo, Jane de Eston Armond, Lucas Melo Neves

**Affiliations:** I Universidade Santo Amaro Programa de Pós-Graduação em Ciências da Saúde São Paulo SP Brasil Universidade Santo Amaro. Programa de Pós-Graduação em Ciências da Saúde. São Paulo, SP, Brasil; II Faculdade de Medicina do ABC Divisão de Ginecologia Endócrina, Menopausa e Planejamento Familiar Departamento de Obstetrícia e Ginecologia São Bernardo do Campo SP Brasil Faculdade de Medicina do ABC. Divisão de Ginecologia Endócrina, Menopausa e Planejamento Familiar. Departamento de Obstetrícia e Ginecologia. São Bernardo do Campo, SP, Brasil; III Universidade de São Paulo Faculdade de Medicina Departamento de Psiquiatria São Paulo SP Brasil Universidade de São Paulo. Faculdade de Medicina. Programa de transtorno bipolar. Departamento de Psiquiatria, São Paulo, SP, Brasil

**Keywords:** Death, Self-Injurious Behavior, Global Health, Epidemiological Studies

## Abstract

**OBJECTIVES:**

To compare rates, disability-adjusted life years (DALYs), and years of healthy life lost due to disability (YLDs) associated with deaths due to self-harm in Brazil.

**METHODS:**

This epidemiological study utilized secondary data obtained from the Global Burden of Disease Study. Analytical examinations were conducted to provide detailed descriptions of national and subnational rates.

**RESULTS:**

We identified mortality rates, DALYs, and YLDs resulting from deaths due to self-harm - national data from 26 states and the Federal District - between 1990 and 2019. The national rates in 1990 and 2019 were the same for mortality = 6.2 deaths per 100.000 inhabitants, reduced for DALYs = 312-289 DALYs, and the same for YLDs = 1.6 YLDs. Four united federations had higher mortality rates, DALYs, and YLDs caused by self-harm compared to national rates throughout the analyzed period (between 1990 and 2019) – Goiás (mortality = 11-67%, DALYs = 13-73%, and YLDs = 4-45%), Mato Grosso do Sul (mortality = 23-42%, DALYs = 28-46%, and YLDs = 13-64%), Minas Gerais (mortality = 5-25%, DALYs = 7-25%, and YLDs = 19-35%), and Rio Grande do Sul (mortality = 73-98%, DALYs = 55-84 %, and YLDs = 52-70%).

**CONCLUSION:**

Although national mortality rates and YLD caused by self-harm have been maintained, there has been a decrease in the incidence of DALYs. However, certain states in Brazil have rates higher than the national average, indicating the need for multiple strategies to be implemented to reduce mortality rates, DALYs, and YLDs resulting from self-harm in these specific states.

## INTRODUCTION

More than 56 million deaths were registered worldwide in 2019, despite 30 years of global health improvements and advances^1^. Although the significance of several causes of death warrant investigation, we highlight death resulting from self-harm. The term “death by self-harm” encompasses a broad spectrum of fatal behaviors, including but not limited to hanging, self-poisoning, engaging in reckless activities, neglecting self-care, and overdosing^2^.

Numerous studies have been conducted to evaluate the incidence of self-harm-related mortality at the continental or national level. Naghavi et al.^3^ conducted a comprehensive analysis of global suicide mortality rates by examining various regions and encompassing data from 195 countries from 1990 to 2016. In a comparable manner, Castelpietra et al.^4^ investigated the prevalence, frequency, and number of years of life lost due to self-harm among young individuals aged 10–24 years in European nations. A recent study by Barbalat and Liu^5^ established a correlation between the quantity of DALYs - disability-adjusted life years: one DALY represents the loss of the equivalent of one year of full health - attributed to self-harm and sociodemographic development. Barbalat and Liu^5^ focused on several geographic locations, including Africa, Southeast Asia, the West Pacific, the Americas, and Europe. However, it is important to emphasize the possibility of comparing different federative units (FUs) within the same nation, as previously described in other studies^6, 7^.

Even though Dávila-Cervantes^8^ looked at Latin America from 1990 to 2019 (comparing countries) and Malta et al.^9^ compared mortality from self-harm in Brazil between 1990 and 2015, no studies have specifically investigated the DALY rates resulting from self-harm within each of the FUs that constitute Brazil. Furthermore, it is worth noting the absence of a comparative analysis on the number of YLDs - years of healthy living lost due to disability: one YLD represents the equivalent of one full year of healthy life lost due to disability or ill health. It is evident that the national rates of deaths, DALYs, and YLDs can vary among the 27 FUs in Brazil, especially because of structural differences among FUs, as well as socioeconomic and demographic characteristics of each FU. Thus, we consider these comparisons to be necessary.

Consequently, the objectives of this study were: a) to compare the rates of deaths, DALYs, and YLDs resulting from self-harm across Brazil at both national and subnational levels (27 FUs) in the years 1990 and 2019, b) to examine the percentage differences in the rates of self-harm deaths, DALYs, and YLDs between national and subnational levels (FUs) from 1990 to 2019, c) to analyze the trends in the rates of deaths, DALYs, and YLDs resulting from self-harm in Brazilian FUs across five age groups (all ages; 10–24; 25–49; 50–74; ≥ 75). Our hypothesis was that national Brazilian rates would exhibit disparities at the subnational level (FUs).

## METHODS

### Data Source

The “Global Burden of Disease Study”, known worldwide as the “GBD study” has become an important format to track mortality rates, DALYs, and YLDs, since the study collects data on global deaths and provides independent population estimates for each of the world’s 204 countries and territories - in some cases with national and subnational data - using a standardized and replicable method^1^. To provide a more precise explanation, the identification of self-harm as the underlying cause of mortality in the GBD study relies on the use of designated codes derived from the International Classification of Diseases (ICD-10 codes X60-X64.9, X66-X84.9, Y87.0; ICD-9 codes E950-E959)^10,11^.

This study used secondary data based on estimated mortality rates from self-harm from the GBD 2019, as previously described completely^1^. Overall, the GBD 2019 estimation process is based on multiple data sources relevant to each illness or injury, including censuses, household surveys, civil registration, vital statistics, disease records, and healthcare usage, as well as air pollution monitors, satellite imagery, disease reports, and other sources^1^. All GBD 2019 data were identified from a systematic review of published studies, searches on government and international organization websites, published reports, primary data sources such as Demographic and Health Surveys, and dataset contributions from GBD contributors, considering 19,354 sources reporting deaths^1^.

### Ethical Approval

This research was conducted following the criteria established by Resolution 466/12 of the National Health Council of Brazil. The data used in this investigation originated from publicly available secondary databases. The databases were collected via the Global Health Data Exchange of the Institute for Health Metrics and Evaluation (IHME).

### Data Search and Refinement Protocol

We used the Global Health Data Exchange (GHDx) tool - (Global Burden of Disease Collaborative Network - Seattle, United States: Institute for Health Metrics and Evaluation [IHME]), 2020, available from https://vizhub.healthdata.org/gbd-results/ .

The GHDx tool allows data to be located according to the organizations involved, geography, period, and content. We considered the following items to select data: locations: Brazil (national level and subnational level – by FUs); years: 1990–2019; age range: all ages; 10–24 years; 25–49 years; 50–74 years; ≥ 75 years; metrics (units): rate per 100,000; measures: deaths, DALYs, and YLDs; sex: both sexes; causes: self-harm.

### Statistical Analysis

The selected data were exported to an Excel spreadsheet using the years 1990–2019 as designated units. In summary, data on mortality rates per 100,000 individuals at national and subnational levels (FUs) and age groups (all ages; 10 to 24 years; 25 to 49 years; 50 to 74 years; ≥ 75 years) were transferred to a spreadsheet. The analysis of specific rates of self-harm deaths, DALYs, and YLDs was the focus of the study: a) National and subnational levels (FUs) were compared only in 1990 and 2019. In this analysis, FUs with mortality rates higher than the national level were highlighted (percentual data); b) the percentage difference in the rates of deaths, DALYs, and YLDs from self-harm between national and subnational levels (FUs) from 1990 to 2019; c) rates of subnational levels (FUs), taking into account different years and age groups (10-24 years, 25–49 years, 50–74 years, and ≥ 75 years, considering all periods (from 1990 to 2019). For this analysis, we considered only all age groups, and the interpretation was considered an increase with a percentage higher than 0% and a decrease with a percentage lower than 0%.

## RESULTS

A total of 340,076 deaths caused by self-harm in Brazil between 1990 and 2019 were identified in GBD data, with annual cases ranging from 8,275 to 16,657 deaths per year (Table). Even the increase in absolute deaths caused by self-harm in Brazil between 1990 and 2019, the mean rate of deaths by 100,000 inhabitants was 6.2 (range from 5.9 to 6.5). A total of 17,053,822 cases of self-harming DALYs in Brazil between 1990 and 2019 were identified in GBD data. Annual cases ranged from 427,491 to 810,812 DALYs per year (Table). Even with the increase in absolute DALYs due to self-harm in Brazil between 1990 and 2019, the rates by 100,000 inhabitants reduced from 312 DALYS in 1990 to 289 DALYs in 2019 (range from 312 to 289). A total of 20,903,448 cases of self-harming YLDs in Brazil between 1990 and 2019 were identified in GBD data, with annual cases ranging from 604,336 to 821,426 YLDs per year (Table). Even with the increase in absolute cases of YLDs caused by self-harm in Brazil between 1990 and 2019, the mean rates by 100,000 habitant was 1.6 (range from 1.4 to 1.6).

**Table:** Total number of deaths, DALYs, and YLDs caused by self-harming in Brazil between 1990 and 2019.

Year	Deaths (95%UI)	Rates (95%UI)	DALYs (95%UI)	Rates (95%UI)	YLDs (95%UI)	Rates (95%UI)
1990	8,275 (8,047–8,519)	6.2 (6.0–6.4)	427,491 (415,272–441,417)	312 (302–323)	604,336 (433,609–802,614)	1.6 (1.1–2.2)
1991	8,531 (8,276–8,780)	6.0 (5.9–6.2)	438,813 (425,258–452,431)	303 (294–313)	613,736 (442,024–815,850)	1.6 (1.1–2.2)
1992	8,859 (8,587–9,121)	6.0 (5.8–6.2)	455,301 (441,377–469,371)	298 (288–308)	622,430 (448,696–828,514)	1.6 (1.1–2.1)
1993	9,302 (9,059–9,577)	6.0 (5.9–6.2)	479,571 (465,440–494,691)	300 (291–310)	630,886 (458,019–841,482)	1.6 (1.1–2.1)
1994	9,530 (9,287–9,812)	6.0 (5.8–6.2)	491,800 (478,659–507,176)	299 (290–308)	639,508 (463,977–856,830)	1.6 (1.1–2.1)
1995	9,606 (9,346–9,891)	5.9 (5.8–6.2)	495,199 (481,014–510,312)	295 (287–307)	648,711 (471,244–864,860)	1.6 (1.1–2.1)
1996	9,495 (9,254–9,785)	5.9 (5.8–6.1)	490,600 (477,215–505,618)	295 (287–306)	656,152 (476,810–876,630)	1.6 (1.1–2.1)
1997	9,551 (9,308–9,795)	6.0 (5.9–6.2)	490,683 (477,552–503,735)	299 (291–306)	660,085 (481,951–881,380)	1.6 (1.1–2.1)
1998	9,807 (9,529–10,071)	6.2 (6.0–6.3)	502,210 (487,108–516,041)	305 (297–313)	662,360 (482,030–885,609)	1.5 (1.1–2.1)
1999	9,659 (9,375–9,948)	6.2 (6.0–6.4)	493,990 (477,326–508,810)	307 (295–316)	664,754 (485,283–887,862)	1.5 (1.1–2.1)
2000	9,795 (9,512–10,090)	6.2 (5.9–6.4)	498,792 (483,048–513,840)	305 (293–314)	669,151 (488,432–893,101)	1.5 (1.1–2.1)
2001	10,256 (9,972–10,552)	6.2 (6.0–6.4)	523,250 (508,658–538,727)	306 (295–314)	675,405 (495,773–901,092)	1.5 (1.0–2.1)
2002	10,383 (10,102–10,711)	6.2 (6.0–6.4)	529,308 (514,687–545,360)	306 (295–314)	682,039 (502,571–904,651)	1.5 (1.0–2.0)
2003	10,461 (10,196–10,779)	6.2 (6.0–6.4)	531,691 (517,601–547,419)	306 (296–314)	688,350 (505,843–917,155)	1.5 (1.0–2.0)
2004	10,696 (10,441–10,985)	6.3 (6.1–6.5)	541,368 (527,252–556,204)	308 (298–318)	694,236 (509,918–923,677)	1.5 (1.0–2.0)
2005	10,940 (10,647–11,246)	6.3 (6.1–6.5)	549,962 (534,405–565,123)	307 (298–316)	699,701 (515,823–929,248)	1.4 (1.0–2.0)
2006	11,905 (10,787–11,420)	6.3 (6.1–6.5)	558,447 (542,844–573,616)	309 (299–317)	701,992 (518,863–931,191)	1.4 (1.0–1.9)
2007	11,435 (11,140–11,754)	6.3 (6.2–6.5)	573,420 (558,796–588,642)	308 (299–316)	700,301 (518,623–929,947)	1.4 (1.0–1.9)
2008	11,767 (11,461–12,073)	6.3 (6.1–6.5)	588,457 (573,787–605,060)	306 (298–314)	696,823 (515,149–929,242)	1.4 (1.0–1.9)
2009	11,998 (11,672–12,322)	6.3 (6.1–6.4)	598,416 (581,911–615,330)	304 (298–311)	695,569 (513,788–929,381)	1.4 (1.0–1.9)
2010	11,979 (11,677–12,282)	6.3 (6.1–6.4)	595,526 (580,134–612,006)	303 (295–310)	699,859 (516,291–931,940)	1.4 (1.0–1.9)
2011	12,364 (12,053–12,685)	6.4 (6.2–6.5)	613,180 (596,229–629,885)	306 (300–314)	710,042 (521,843–940,042)	1.5 (1.0–2.0)
2012	12,691 (12,349–13,027)	6.4 (6.2–6.5)	625,397 (608,764–642,272)	307 (300–314)	723,348 (532,767–960,216)	1.5 (1.0–2.0)
2013	12,938 (12,620–13,269)	6.4 (6.2–6.6)	636,453 (619,988–652,773)	306 (299–315)	738,010 (542,968–977,710)	1.5 (1.1–2.1)
2014	13,164 (12,846–13,495)	6.4 (6.2–6.6)	646,668 (631,833–662,979)	305 (297–315)	752,911 (554,599–998,920)	1.5 (1.1–2.1)
2015	13,651 (13,304–14,008)	6.4 (6.2–6.7)	666,518 (650,680–684,000)	304 (296–317)	766,395 (562,486–101,638)	1.6 (1.1–2.1)
2016	14,441 (14,083–14,824)	6.5 (6.4–6.8)	704,287 (687,164–723,498)	309 (301–320)	779,762 (572,439–103,537)	1.5 (1.1–2.1)
2017	15,056 (14,669–15,409)	6.4 (6.2–6.7)	732,750 (714,580–751,549)	302 (292–316)	795,128 (579,184–105,384)	1.5 (1.0–2.0)
2018	15,697 (15,314–16,095)	6.3 (6.0–6.7)	763,462 (744,922–782,354)	294 (282–314)	810,042 (590,092–107,563)	1.5 (1.1–2.1)
2019	16,657 (16,236–17,048)	6.2 (5.9–6.8)	810,812 (791,528–829,859)	289 (274–315)	821,426 (596,720–108,704)	1.6 (1.2–2.2)

UI: uncertainty interval; DALYs: disability-adjusted life years - one DALY represents the loss of the equivalent of one year of full health; YLDs: years of healthy living lost due to disability - one YLD represents the equivalent of one full year of healthy life lost due to disability or ill health.

**Figure 1:**
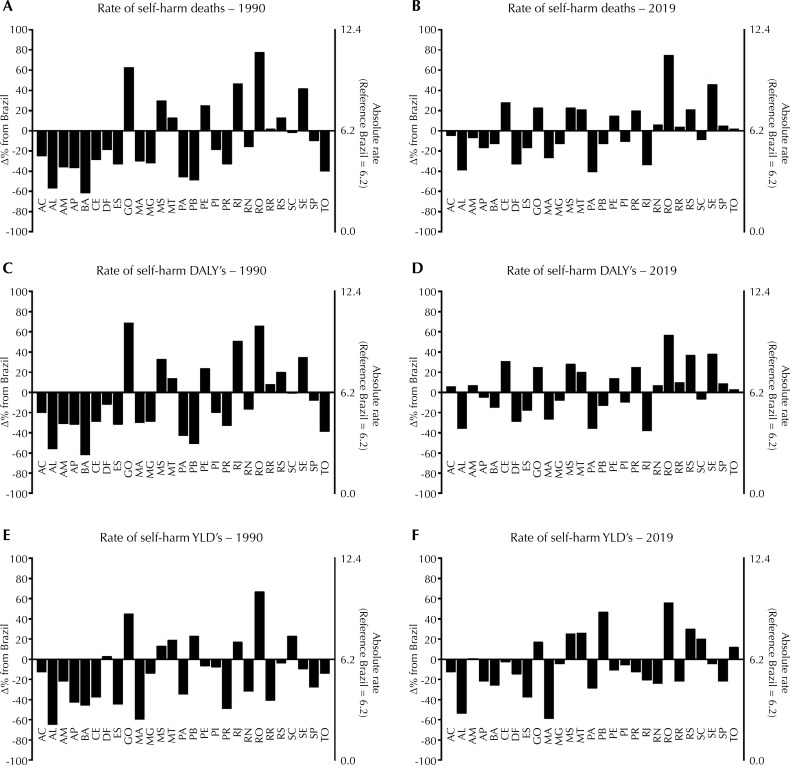
Rates (per 100,000 habitants) of self-harm deaths (Panel A and B), disability-adjusted life Years - DALYs (Panel C and D), and years of healthy living lost due to disability - YLDs (Panel E and F) in each Brazilian Federative Unit in 1990 and 2019. AC (Acre), AL (Alagoas), AM (Amazonas), AP (Amapá), BA (Bahia), CE (Ceará), DF (Distrito Federal), ES (Espírito Santo), GO (Goiás), MA (Maranhão), MG (Minas Gerais), MS (Mato Grosso do Sul), MT (Mato Grosso), PA (Pará), PB (Paraíba), PE (Pernambuco), PI (Piauí), PR (Paraná), RJ (Rio de Janeiro), RN (Rio Grande do Norte), RO (Rondônia), RR (Roraima), RS (Rio Grande do Sul), SC (Santa Catarina), SE (Sergipe), SP (São Paulo), TO (Tocantins).

Figure 1 presents the rates of self-harm deaths, DALYs, and YLDs (percentual and absolute data) comparing only the years 1990 and 2019 in Brazil and in the FUs.

For rates of self-harm deaths in 1990 (Panel A) in Distrito Federal and 8 states (Goiás, Mato Grosso, Mato Grosso do Sul, Pernambuco, Rio de Janeiro, Roraima, Rio Grande do Sul, and Sergipe) ranged from 2 to 78% - presented higher rates compered national rate. In 2019 (Panel B) 8 states (Ceará, Goiás, Mato Grosso, Mato Grosso do Sul, Paraná, Rio Grande do Norte, Rondônia, Roraima, Rio Grande do Sul, Sergipe, São Paulo, and Tocantins) range from 2 to 75% - presented higher rates than the national rate.

For rates of DALYs, in 1990 (Panel C) 9 states (Goiás, Mato Grosso, Mato Grosso do Sul, Pernambuco, Rio de Janeiro, Rondônia, Roraima, Rio Grande do Sul, and Sergipe) ranged from 8 to 69% - presented higher rates compared with the national rate. In 2019 (Panel D), 15 states (Acre, Amazonas, Ceará, Goiás, Mato Grosso, Mato Grosso do Sul, Pernambuco, Paraná, Rio Grande do Norte, Rondônia, Roraima, Rio Grande do Sul, Sergipe, São Paulo, and Tocantins) ranged from 3 to 57% - presented higher rates than the national rate.

For rates of YDL’s, in 1990 (Panel E) Distrito Federal and 8 states (Goiás, Mato Grosso, Mato Grosso do Sul, Paraíba, Rio de Janeiro, Rondônia, and Santa Catarina) ranged from 3 to 67% - presented higher rates compered national rate. In 2019 (Panel F) 8 states (Goiás, Mato Grosso, Mato Grosso do Sul, Paraíba, Rondônia, Rio Grande do Sul, Santa Catarina, and Tocantins) ranged from 1 to 56% - presented higher rates than the national rate.

**Figure 2:**
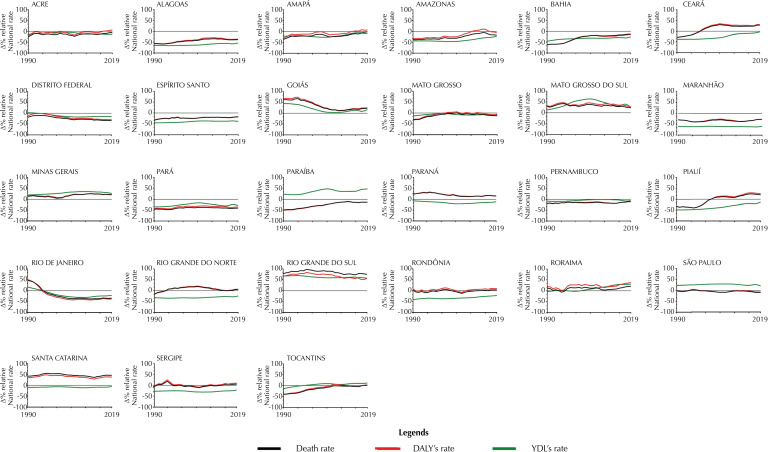
Rates (per 100,000 habitants) of self-harm deaths, DALYs, and YLDs in each federal unit of Brazil in 1990 and 2019. Data are presented as the percentage of difference in the rates of deaths, DALYs, and YLDs from self-harm at national and subnational levels (FUs) from 1990 to 2019.

Figure 2 presents the rates of self-harm deaths, DALYs, and YLDs (percentual data) comparing all years (between 1990 and 2019) considering the mean of Brazil and the mean of each FU. A total of 4 FUs (Goiás, Mato Grosso do Sul, Minas Gerais, Rio Grande do Sul) presented percentage differences in rates of self-harm deaths, DALYs, and YLDs compared with the national level in all periods (between 1990 and 2019).

### Trends in mortality due to self-harm mortality in Brazilian states from 1990 to 2019 by age group

**Figure 3:**
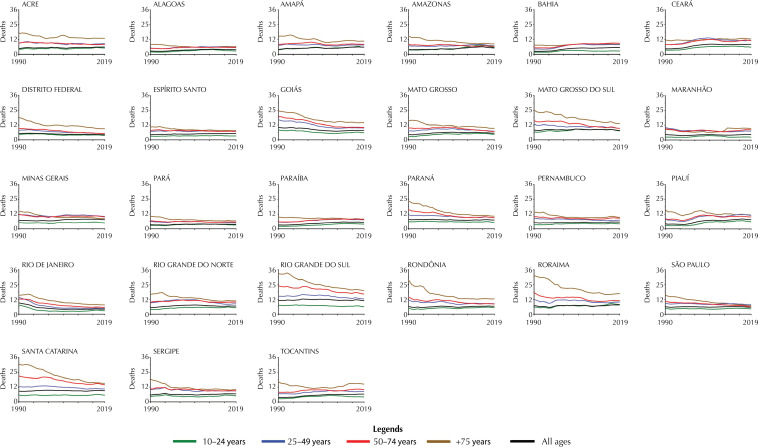
Trends of Rates (per 100,000 habitants) of self-harm deaths of Brazil and in each Brazilian Federative Unit. Data are presented per year (from 1990 to 2019) and per age group (10–24 years; 25–49 years; 50–74 years; ≥ 75 years; all ages).

Figure 3 presents the rates of self-harm deaths in Brazil and at the subnational level (FUs) categorized by age group, including 10–24 years, 25–49 years, 50–74 years, ≥ 75 years, and all ages. For Brazil rates of self-harm deaths per age group between 1990 and 2019, higher rates were observed in the group ≥ 75 years (9.7 to 15.8), followed by the 50–74 years group (8.5 to 11.7), 25–49 years group (8.2 to 10.0), and 10–24 years group (4.2 to 4.8). Considering subnational data, only 6 FUs presented predominance of ≥ 75 years group, follow 50–74 years group, 25–49 years group, and 10–24 years group - Amapá, Goiás, Pernambuco, Rio Grande Do Sul, Roraima, and Santa Catarina.

### Trends in disability-adjusted life years due to death by self-harm in Brazilian states from 1990 to 2019 by age group

**Figure 4:**
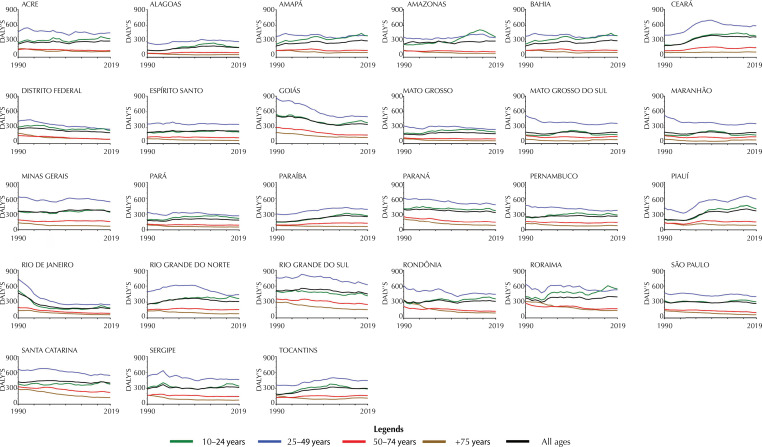
Trends of Rates (per 100,000 habitants) of self-harm DALYs (Disability Adjusted Life Years) in Brazil and in each Brazilian Federative Unit. Data are presented per year (from 1990 to 2019) and per age group (10–24 years; 25–49 years; 50–74 years; ≥ 75 years; all ages).

Figure 4 presents the rates of DALY self-harm in Brazil and at the subnational level (FUs) categorized by age group, including 10–24 years, 25–49 years, 50–74 years, ≥ 75 years, and all ages. For Brazil rates of DALYs per age group between 1990 and 2019, higher rates were observed in the group 25–49 years (428.1 to 530.9), follow by 10–24 years group (289.4 to 335.7), 50–74 years group (136.8 to 186.4), and the +75-year-old group (78.0 to 142.2). Considering subnational data, all FUs exhibited a predominance of 25–49 years group.

### Trends in the number of years of healthy life lost due to death by self-harm in Brazilian states from 1990 to 2019

**Figure 5:**
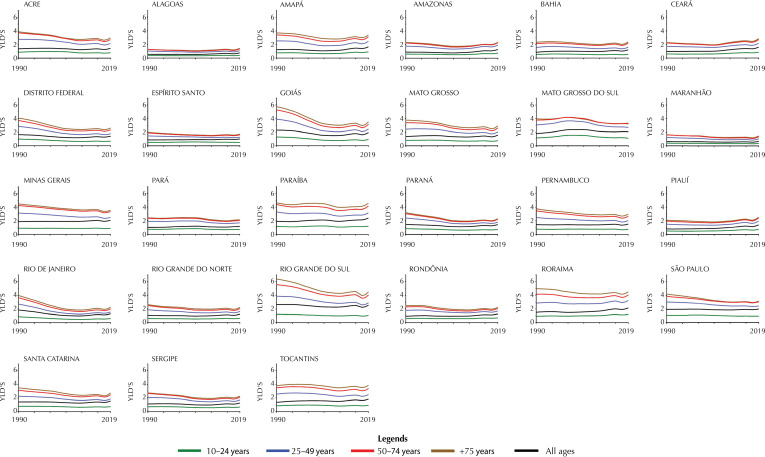
Trends of Rates (per 100,000 habitants) of self-harm YLDs (Years of healthy life lost due to disability) in Brazil and in each Brazilian Federative Unit. Data are presented per year (from 1990 to 2019) and per age group (10–24 years; 25–49 years; 50–74 years; ≥ 75 years; all ages).

Figure 5 presents the rates of self-harm YLDs by age group (10–24 years; 25–49 years; 50–74 years; ≥ 75 years; all ages). According to the data, individuals aged ≥ 75 years exhibit a greater rates of self-harm YLDs. For Brazil rates of self-harm YLDs per age group between 1990 and 2019, higher rates were observed in the group ≥75 years (2.6 to 3.7); follow by 50–74 years group (2.5 to 3.5), 25–49 years group (1.9 to 2.6), and 10–24 years group (0.7 to 0.9). Considering subnational data, only 1 FU not presented predominance of ≥ 75 years group - Mato Grosso do Sul.

## DISCUSSION

The present study analyzed the temporal changes in the rates of deaths, DALYs, and YLDs caused by self-harm in the Brazilian population at the national and sub-national levels. Compared with 1990 and 2019, the national death rate was 6.2 deaths per 100,000 inhabitants, the DALY rate decreased from 312 to 289, and the YLD rate was 1.6. When comparing the national data with those of all other FUs, it was found that four FUs—Goiás, Mato Grosso do Sul, Minas Gerais, and Rio Grande do Sul—consistently had higher rates of self-harm deaths, DALYs, and YLDs from 1990 to 2019. When examining age groups, the rates of self-harm deaths at the national level were highest among individuals aged 75 years and older, followed by the 50–74, 25–49, and 10–24 years age groups. The rates of DALYs at the national and FU levels were highest among individuals aged 25–49 years, followed by the 10–24 years, 50–74 years, and individuals aged 75 years and older. The rates of YLDs at the National and FUs were higher for the group aged +75 years, followed by the 50–74 years, 25–49 years, and 10–24 years groups, except for the Mato Grosso do Sul FU group, which exhibited the predominance of the 50–74 years group.

Although the rates of deaths and YLDs from self-harming in Brazil did not change between 1990 and 2019, and the rates of DALYs were reduced, these findings may be viewed as positive. However, the examination of trends by FUs clearly revealed crucial differences. Barbalat and Liu^5^ reported that in some parts of the world, like Europe, countries with higher levels of development have higher rates of self-harm. However, in other parts of the world, like Southeast Asia, countries with greater development have lower rates of self-harm. If we consider economic activity, the majority of FUs in 1990 with a higher mortality rate than the national rate were richer FUs (group of the 50% with a higher gross domestic product - GDP) in Brazil (except FU Rondônia), which could suggest the impact of economic activity on this rate.

However, 2019 data show an increase in FUs, mostly among the poorest (all of which have a GDP 50% lower) in Brazil. Barbalat and Liu^5^ identified a link between self-harm and social and economic progress in some places and times but not everywhere. The GBD 2019 Diseases and Injuries Collaborators^1^ found that global DALYs remained almost constant between 1990 and 2019, and Brazil’s self-harming DALY reduction is encouraging.

The predominance of individuals aged ≥ 75 years exhibiting a greater rate of self-harm deaths, DALYs, and YLDs can be explained by the ideas of Tan and Cheung^12^, who showed that older age leads to a significant increase in self-harm. Additionally, other factors that present a positive correlation between mental disorders, such as dementia, and the prevalence of suicide in older adults have already been described^13^, as well as associations of common factors verified in older adults, such as previous and current psychiatric treatment and single and living alone^14^.

The FUs of Goiás, Mato Grosso do Sul, Minas Gerais, and Rio Grande do Sul always differed from the national rates (between 1990 and 2019) and were the most significant findings of our study. Our goal was to identify FUs with significant differences from those in Brazil and not to explain this finding. However, demographic factors (youth, female sex, socioeconomic disadvantage, and homosexual or bisexual orientation)^2^ and social and family environment factors (adverse childhood experiences and interpersonal difficulties during adolescence)^2^ may be linked to self-harm. Importantly, all Brazilian FUs exhibit the effects of the conditions listed. Therefore, explaining these rate differences is a multifaceted problem that our data cannot solve. Our findings indicate an unusual value for health system leadership in this area, emphasizing the need for self-harm actions in these functional units.

The primary merit of this study is its ability to provide comprehensive estimations of self-harm death rates, DALYs, and YLDs in Brazil at both national and subnational levels. These estimations cover the period from 1990 to 2019 and are calculated using known procedures, ensuring comparability over time.

Our research is not devoid of limitations. Although we found an elevated incidence of fatalities due to self-harm in some FUs, our study only describes the features of these cases and does not provide an explanation for the underlying causes of this phenomenon. Additionally, our study did not analyze different types of deaths, DALYs and YLDs, by self-harming, self-poisoning, engaging in reckless activities, neglecting self-care, and overdosing.

In conclusion, when comparing 1990 and 2019, the national mortality and YLD rates remained unchanged, whereas the DALY rates decreased. From 1990 to 2019, the states of Goiás, Mato Grosso do Sul, Minas Gerais, and Rio Grande do Sul consistently exhibited higher rates of self-harm deaths, DALYs, and YLDs than the national rates. These data indicate the significance of employing various strategies to decrease the mortality rate, DALYs, and YLDs via self-harm in some FUs in Brazil.

## References

[1] Vos T, Lim SS, Abbafato C, Abbas KM, Abbasi M, Abbasifard M (2020). Global burden of 369 diseases and injuries in 204 countries and territories, 1990-2019: a systematic analysis for the Global Burden of Disease Study 2019. Lancet.

[2] Skegg K, Self-harm SK (2005). Lancet.

[3] Naghavi M (2019). Global, regional, and national burden of suicide mortality 1990 to 2016: systematic analysis for the Global Burden of Disease Study 2016. BMJ.

[4] Castelpietra G, Knudsen AK, Agardh EE, Armocida B, Beghi M, Iburg KM (2022). The burden of mental disorders, substance use disorders and self-harm among young people in Europe, 1990-2019: fndings from the Global Burden of Disease Study 2019. Lancet Reg Health Eur.

[5] Barbalat G, Liu S (2022). Socio-demographic development and burden of mental, substance use disorders, and self-harm: an ecological analysis using the Global Burden of Disease study 2019. Aust N Z J Psychiatry.

[6] Zhou M, Wang H, Zhu J, Chen W, Wang L, Liu S (2016). Cause-specific mortality for 240 causes in China during 1990-2013: a systematic subnational analysis for the Global Burden of Disease Study 2013. Lancet.

[7] Abtahi M, Koolivand A, Dobaradaran S, Yaghmaeian K, Khaloo SS, Jorfi S (2018). National and subnational mortality and disability-adjusted life years (DALYs) attributable to 17 occupational risk factors in Iran, 1990-2015. Environ Res.

[8] Dávila-Cervantes CA (2022). Suicide burden in Latin America, 1990-2019: findings from the global burden of disease study 2019. Public Health.

[9] Malta DC, Minayo MC, Soares AM, Silva M, Montenegro MM, Ladeira RM (2017). Mortality and years of life lost by interpersonal violence and self-harm: in Brazil and Brazilian states: analysis of the estimates of the Global Burden of Disease Study, 1990 and 2015. Rev Bras Epidemiol.

[10] International Facility Management Association Evaluation IfHMa: about Dataset Records 2024.

[11] Murray CJ (2022). The Global Burden of Disease Study at 30 years. Nat Med.

[12] Tan YM, Cheung G (2019). Self-harm in adults: a comparison between the middle-aged and the elderly. N Z Med J.

[13] Kulak-Bejda A, Bejda G, Waszkiewicz N (2021). Mental disorders, cognitive impairment and the risk of suicide in older adults. Front Psychiatry.

[14] Troya MI, Babatunde O, Polidano K, Bartlam B, McCloskey E, Dikomitis L (2019). Self-harm in older adults: systematic review. Br J Psychiatry.

